# Hydration Status as a Predictor of High-altitude Mountaineering Performance

**DOI:** 10.7759/cureus.918

**Published:** 2016-12-07

**Authors:** Eric Ladd, Katherine M Shea, Patrick Bagley, Paul S Auerbach, Elizabeth A Pirrotta, Ewen Wang, Grant Lipman

**Affiliations:** 1 Department of Emergency Medicine, Stanford University School of Medicine; 2 University of New England College of Osteopathic Medicine, University of New England; 3 School of Medicine, Stanford University

**Keywords:** performance, hydration, mountaineering, ultrasound, dehydration

## Abstract

Background: Hydration status is a controversial determinant of athletic performance. This relationship has not been examined with mountaineering performance.

Methods: This was a prospective observational study of mountaineers who attempted to climb Denali in Alaska. Participants’ urine specific gravity (SG), and ultrasound measurements of the inferior vena cava size and collapsibility index (IVC-CI) were measured at rest prior to ascent. Upon descent, climbers reported maximum elevation gained for determination of summit success.

Results: One hundred twenty-one participants enrolled in the study. Data were collected on 111 participants (92% response rate); of those, 105 (87%) had complete hydration data. Fifty-seven percent of study participants were found to be dehydrated by IVC-CI on ultrasound, and 55% by urine SG. No significant association was found with summit success and quantitative measurements of hydration: IVC-CI (50.4% +/- 15.6 vs. 52.9% +/- 15.4, p = 0.91), IVC size (0.96 cm +/- 0.3 vs. 0.99 cm +/- 0.3, p = 0.81), and average SG (1.02 +/- 0.008 vs. 1.02 +/- 0.008, p = 0.87). Categorical measurements of urine SG found 24% more successful summiters were hydrated at 14 Camp, but this was not found to be statistically significant (p = 0.56). Summit success was associated with greater water-carrying capacity on univariate analysis only: 2.3 L, 95% confidence interval (2.1 – 2.5) vs. 2.1 L, 95% confidence interval (2 – 2.2); p < 0.01.

Conclusions: Intravascular dehydration was found in approximately half of technical high-altitude mountaineers. Hydration status was not significantly associated with summit success, but increased water-carrying capacity may be an easy and inexpensive educational intervention to improve performance.

## Introduction

Mountain climbing is a popular pastime, with over 120 million annual visitors to the European Alps [1] and 25,000 annual climbers attempting Mount Kilimanjaro [2].  Much research has been done on the effects of nutrition and acclimatization on high-altitude exercise capacity, but little study has focused on hydration and performance in the high mountains. At high altitudes the body has a natural tendency to have increased insensible water loss [3], and in alpine environments where snow must be melted for drinking water, time and fuel resources may limit optimal hydration. Dehydration has been shown to degrade high-altitude aerobic capacity [4]. There has been some debate on the effect dehydration has on performance in endurance athletes. Observational studies have found greater weight loss (more dehydration) does not correlate with poorer performance [5]. In fact, the fastest athletes in races have often been found to lose the greatest amount of weight during competition [6]. These findings contrast with controlled prospective studies that show dehydration decreases athletic performance [7]. Furthermore, dehydration has been linked with increased physiologic strain and increased perceived exertion [8]. Hydration and performance trends from low-altitude endurance events have limited generalizability to the hypobaric, cold-weather environments found in the high mountains where the majority of travelers are dehydrated [9]. The role that dehydration plays in mountaineering performance has yet to be explored.

Hydration status is challenging to measure, with poor diagnostic accuracy of common clinical symptoms and signs [10]. A simple, indirect method of estimating hydration status is by analysis of urine SG, which correlates well with urine osmolality [11] and is recommended for measuring hydration status in athletes [12]. A person who is otherwise healthy will concentrate his or her urine when dehydrated, in order to retain free water, compared to an individual in a euhydrated state. Another objective measurement of hydration status is by ultrasound measurement of the IVC-CI during the respiratory cycle. This allows direct measurement of the vascular status and has been shown to reliably predict volume status in both emergency department patients and healthy cohorts in field clinics [13]. During inspiration, generated negative intrathoracic pressure and positive intra-abdominal pressure increases venous return and collapses the IVC. Dehydrated (hypovolemic) individuals have a larger degree of collapse than those who are euvolemic. It is well accepted that a collapse of the IVC by 50% or more on inspiration is associated with a depleted intravascular state [14]. Intravascular hydration measurements by ultrasound have previously not been attempted in a technical alpine hypobaric environment.

The objective of this study was to prospectively evaluate hydration status by two objective measures, urine SG and IVC-CI on ultrasound in climbers attempting to ascend Denali, and correlate their hydration status with mountaineering performance.

## Materials and methods

Participants

There are approximately 1,200 climbers who annually attempt to summit Denali, Alaska, [15] the tallest mountain in North America at 6,168 m (20,237 ft). The majority of these climbers attempt the West Buttress route and in doing so, pass through the 4,267 m (14,000 ft) camp (14 Camp). This study was conducted between June 12 and July 5, 2013, at 14 Camp. Inclusion criteria were healthy male and female climbers between the ages of 18 and 65 years. Exclusion criteria included past medical history of significant cardiopulmonary derangement, severe anemia, Raynaud’s disease, peripheral vascular disease, or suspected pregnancy. Other exclusion criteria were climbers who took more than 10 days to reach 14 Camp, sleeping elevation greater than 4,276 m (14,000 ft) within the preceding week, and being at 14 Camp for more than 24 hours prior to enrollment. Subjects participated on a voluntary basis and received no financial compensation. The study was approved by the Stanford University School of Medicine Institutional Review Board (IRB-25813) and the Denali National Park Service (NPS). Patient informed consent was obtained.

Study site and logistics

A flyer advertisement was distributed by the NPS to all registered climbers prior to their climb. Climbers were informed of the study as they checked into the NPS ranger station in Talkeetna, Alaska (106 m or 348 ft) during their orientation video, which is mandatory for all registered climbers. Potential participants were instructed to check in at the study tent situated on the glacier at 14 Camp within 24 hours of their initial arrival. The majority of climbers arriving at 14 Camp shuttle a gear load from 3,115 m (11,200 ft), then return to a lower elevation to sleep. This practice is termed a “carry” and is part of the acclimatization process. This carry was not an exclusion criteria, and the 24-hour time limit for study inclusion began on their subsequent arrival at 14 Camp.

Study participants were seated in the research tent and completed a demographics questionnaire and Lake Louise Questionnaire (LLQ), a well-validated self-report questionnaire on acute mountain sickness (AMS) symptoms. LLQ score greater than or equal to three and presence of headache is considered positive for AMS [16]. Demographic data included age, sex, whether or not the subject used a paid guide, whether the subject was currently working as a professional guide, prior history of altitude illness, use of acetazolamide or ibuprofen, the participants’s altitude of residence, the number of days they took to reach 14 Camp, and the number of hours they had been at 14 Camp at the time of testing. After survey completion, baseline vital signs were obtained including peripheral oxygen saturation (SpO2), heart rate, and respiratory rate. Participants were blinded to their physiologic measures so as to not artificially increase their oxygenation by hyperventilating or pressure breathing. Participants subsequently underwent IVC measurement and provided a urine sample. Water-carrying capacity was defined by the total amount of water that could be carried by a climber. For example, if a climber had two, one-liter water bottles, they had a two-liter water-carrying capacity. After pre-climb testing, study participants proceeded to climb toward the summit at their own pace (usually within three to seven days of testing). The climb from 14 Camp to the summit often takes approximately two to three days in good weather. Upon descent, climbers were asked to check in at the study tent to complete a survey regarding the outcome of their summit attempt with maximum elevation gained, their reason for turn-around if they failed to summit, and the worst altitude symptoms they experienced above 14 Camp by LLQ. For participants who did not provide this information, an e-mail survey (SurveyMonkey, Palo Alto, CA) and two follow-up reminder e-mails were sent. If the e-mails were not responded to, the participant was telephoned once.

Equipment

**Figure 1:**
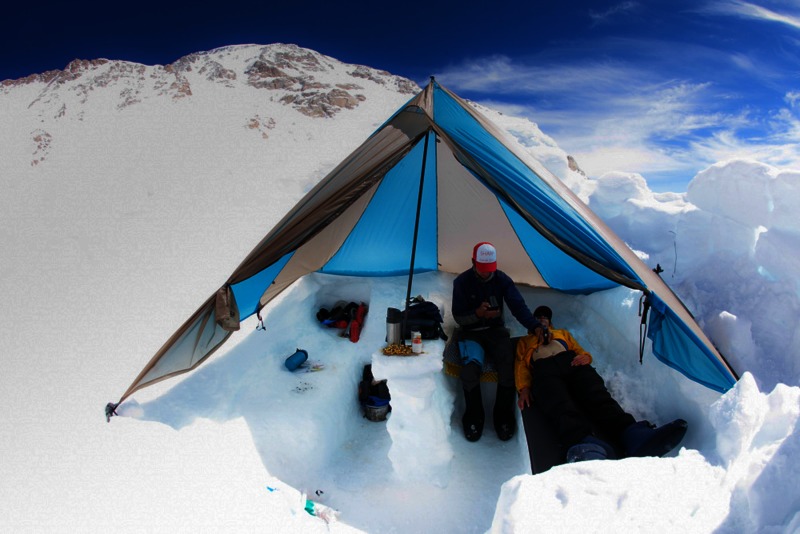
Study enrollment and ultrasound data acquisition site

Participants lay in a recumbent position for five minutes prior to IVC ultrasound measurements on a snow bench insulated with a thin, generic foam pad under an open dome tent (Figure 1). Inferior vena cava measurements were made with a hand-held, 11.6 oz, MobiUS™ SP1 ultrasound system (Mobisante, Redmond, WA) with a 7.5 MHz vascular probe. The technique to measure IVC and IVC-CI has been well described in the literature [17]. In brief, the ultrasound probe was placed parallel to the xiphoid process over the right upper quadrant in the longitudinal plane (probe marker-oriented cephalad). Inferior vena cava collapsibility was measured approximately 2 cm from the right atrium and calculated as the relative decrease in the IVC diameter during one respiratory cycle [(expiratory IVC – inspiratory IVC / expiratory IVC) x 100]. Images were saved for off-line caliper measurements by a single reviewer. The single ultrasonographer was blinded to the participants’ demographics questionnaire, AMS status, and urine SG. The ultrasonographer was a senior resident in emergency medicine and met the American College of Emergency Physicians standards for ultrasonographic competency [18].

Oxygen concentration was measured by a Nonin Onyx fingertip pulse oximeter (Nonin Medical Products, Minneapolis, MN). Participants provided a urine sample that was collected in a small, disposable cup and immediately tested for urine SG with Siemens Multistix 10 SG reagent strips (Siemens, Munich, Germany). These test strips are discrete to t 0.005 increments. A three-level classification of hydration status was determined for each participant: hydrated (urine SG = 1.005, 1.010), borderline (urine SG = 1.015), or dehydrated (urine SG = 1.020, 1.025, 1.030). A binary categorization for qualification of hydration status was determined by combining hydrated and borderline criteria into a single category versus dehydration [19].

Statistical analysis

Mountaineering performance was qualified by whether or not the climber reached the summit of Denali.  Summit success was defined as a participant who obtained the maximum elevation of 6,168 m (20,237 ft), and non-summiters were those who failed to reach this elevation. For data with a normal distribution, means and standard deviations (SD) with 95% confidence intervals (CI) are presented. An independent two-sample t-test was used to compare the means of continuous variables, and chi-square analysis was used to compare proportions of categorical variables. Urine SG was analyzed as a continuous variable and then utilized to qualify hydration state as a categorical variable. Variables identified as significant by univariate analysis were entered into a multivariate regression model to predict the outcome variable of successful summiting, controlling for age, sex, and whether or not the subject was a guide. Reported tests of significance are two-tailed, with α = 0.05. All analysis was by SAS 9.4 (SAS Institute, Inc., Cary, NC).

## Results

In the summer of 2013, 1,151 climbers attempted Denali, 1,071 of them by the West Buttress route. There was a 68% success rate of all climbers for summiting on the West Buttress. One hundred twenty-one climbers were enrolled in the study. Forty-six (38%) participants did not return the post-ascent survey and were subsequently contacted using survey software. Thirty-three (72%) of these individuals responded. The remaining 13 subjects were telephoned, of whom three responded. The overall response rate was 111 of 121 (92%), with 67 (60%) successful summiters. Complete hydration data that was used for primary outcome analysis was available on 105 (87%), with summit success in 64 (61%). Participant characteristics are described in Table 1. Non-summiters were significantly more likely to have a prior history of altitude illness, took a longer time to reach 14 Camp, and had less water-carrying capacity. However, this statistical significance was not seen when adjusted on multivariate analysis. The most common water-carrying capacity by the participants was two liters, seen in 58% (n = 39) of successful summiters versus 75% (n = 33). It was more common for successful summiters to carry greater amounts of water.

**Table 1:** Characteristics in summiters and non-summiters * = statistically significant

	*Summiters * Mean (%) N=67 (60%)	(95% CI)	*Non-Summiters* Mean (%) N=44 (40%)	(95% CI)	p value
Age (years)	36	(33.8 – 39.1)	40	(35.9 – 43.3)	0.15
Sex (male)	57 (85%)	(74% – 93%)	32 (73%)	(57% – 85%)	0.11
Guided	33 (49%)	(37% – 61%)	25 (57%)	(29% – 58%)	0.58
Previous altitude illness	8 (12%)	(5% – 22%)	13 (30%)	(17% – 46%)	0.02*
Acetazolamide use	8 (12%)	(5% – 22%)	7 (16%)	(7% – 30%)	0.55
Ibuprofen use	11 (16%)	(8% – 27%)	10 (23%)	(11% – 38%)	0.41
Altitude of residence, m (SD)	754 (819)	(555 – 954)	550 (769)	(316 – 783)	0.19
Days to reach 14 Camp (SD)	6.5 (1.6)	(6.1 – 6.9)	7.2 (1.4)	(6.8 – 7.7)	0.02*
Number of hours at 14 Camp (SD)	17 (7.5)	(15.2 – 18.9)	16 (8.7)	(13.2 – 18.6)	0.46
Carrying capacity, L (SD)	2.3 (0.7)	(2.1 – 2.5)	2.1 (0.4)	(2 – 2.2)	< 0.01*
AMS	29 (43)	(31 – 56)	15 (34)	(20 – 51)	0.46

Fifty-seven percent of study participants were found to be dehydrated by IVC-CI measurements. Similarly, 55% of participants were dehydrated by urine SG. Approximately 80% were dehydrated by either measure, and 34% of participants were dehydrated by both measures of dehydration. No statistically significant differences were found with summit success and the average urine SG, IVC diameter (minimum, maximum, and delta), or IVC-CI (Tables 2-3).

**Table 2:** Hydration measurements by ultrasound and urine specific gravity by summit success IVC = inferior vena cava

	*Summiters * Mean (SD) N=64		(95% CI)	*Non-Summiters* Mean (SD) N=41	(95% CI)	p value
Urine specific gravity	1.020 (0.008)		(1.018 – 1.021)	1.021 (0.008)	(1.018– 1.023)	0.87
IVC maximum (cm)	1.91 (0.3)		(1.83 – 1.99)	1.89 (0.39)	(1.78 – 2.01)	0.29
IVC minimum (cm)	0.95 (0.4)	(0.87 – 1.04)		0.91 (0.37)	(0.79 – 1.02)	0.71
IVC delta (cm)	0.96 (0.3)	(0.88 – 1.04)		0.99 (0.31)	(0.89 – 1.08)	0.81
IVC collapsibility index in %	50.4 (15.6)	(47 – 54)		52.9 (15.4)	(48 – 58)	0.91

**Table 3:** Hydration status by urine specific gravity and summit success Hydrated (USG = 1.005, 1.010), borderline (USG = 1.015), and dehydrated (USG = 1.020, 1.025, 1.030)

Summit success Total	Dehydrated N (%) 58 (55%)	Borderline N (%) 23 (22%)	Hydrated N (%) 24 (23%)	Total N (%) 105
Yes	33 (57%)	16 (70%)	15 (62%)	64 (61%)
No	25 (43%)	7 (30%)	9 (38%)	41 (39%)

## Discussion

Approximately half of the mountaineers in the study were found to be dehydrated by two objective measures of hydration status. No statistically significant difference was seen in those with and without summit success. Urine SG testing showed that almost a quarter more of successful climbers were hydrated at 14 Camp, although this did not reach statistical significance. Greater hydration in this cohort and subsequent summit success may have been due to the greater water-carrying capacity (the same amount that they had to the summit); however, as only a 0.2 liter difference from non-summiters was found, it is of questionable association and clinical significance.

Optimal mountaineering performance may be influenced by many factors including weather, route conditions, team dynamics, physical fitness, and mental determination. Hydration likely plays a role in successful high-altitude mountaineering because the body experiences fluid shifts when dehydrated that result in increased cardiovascular strain as plasma volume declines [20]. Impaired cardiovascular function leads to diminished cutaneous blood flow and subsequently a diminished ability to dissipate heat to the environment, [21] which can occur from high-exertion activities even in cold alpine conditions. Along with physiologic effects, dehydration can negatively impact mental performance [22], an important dimension in technical mountaineering. Furthermore, the hypoxia at high altitude is known to increase fluid losses and contribute to dehydration, which at altitude has been shown to further degrade aerobic capacity [4]. Thus, the subtle hydration differences seen at 14 Camp, if persistent at higher elevations on the mountain, may have contributed to subsequent mountaineering success.

Ultrasound is often espoused as a cutting-edge device to aid clinical diagnosis in the wilderness environment, and it is currently taught that hand-held portable ultrasound can assist the medical practitioner in extreme environments for diagnosis and risk stratification. Ultrasonography has been attempted as a clinical prognosticator at high altitudes [23] but with limited diagnostic success as a point estimate of AMS [24]. Similar to other high-altitude ultrasound studies [13], our results indicated that a single measurement of intravascular volume has limited applicability and is not a useful clinical adjunct to the evaluation and performance determinant of high-altitude mountaineers.

AMS, a constellation of symptoms that range from uncomfortable to debilitating, was seen in over one-third of all study participants who attempted to climb Denali, but this was not associated with hydration status. Dehydration has been hypothesized as a risk factor for AMS [4,25]. It is theorized that dehydration may contribute to the development of AMS as dehydration causes an increased reabsorption of sodium and water by the kidneys, which also causes bicarbonate retention. This increase in bicarbonate retention will limit the body’s compensatory respiratory alkalosis caused by the hypoxic response to the lower partial pressure of oxygen at high altitude. The largest observational study showed an inverse relationship between water intake per day and the development of AMS [9], while another showed only a weak correlation between urine SG with AMS incidence. Both of these studies looked at multiple other hydration variables and did not find significance when correlated with AMS. While dehydration may contribute to AMS, it could also decrease a climber’s chances of reaching the summit by contributing to a sense of exhaustion [26], with subsequent slower ascent rate that could increase a climber's exposure to weather and treacherous alpine conditions. Of note, overhydration is not recommended to prevent AMS [27], and could potentially lead to fatal dilutional hyponatremia.

While summit success is a well-accepted performance measure in mountaineering, it lacks accuracy because there are many environmental factors in the high mountains that may interfere with individual exercise performance. We attempted to account for these external variables by querying the reason for turn-around on the post-ascent survey, but anecdotally we know there was reporting bias by unsuccessful summiters. Other limitations include potential for a referral bias innate to the study design, as climbers feeling poorly upon arrival at 14 Camp were less likely to enroll in the study, but we attempted to counter this by offering all the opportunity to join. The cold stress and hypobaric hypoxia at 14 Camp could have led to a decrease in central venous volume (via altitude-induced and cold-induced diuresis), and exercise and sweat losses may have caused intracellular water loss (but this would be unlikely to have a large effect on plasma volume). We attempted to control for this by gathering information on time spent in 14 Camp prior to measurements, and as there were no significant differences, these hypothetical changes unlikely skewed the results. Hydration status at 14 Camp was not necessarily reflective of hydration status at higher altitudes, and a point estimate was less ideal than multiple measurements of hydration; however, longitudinal measurements were not logistically feasible. While urine SG is generally accepted in emergency departments as a surrogate measure for hydration, the accuracy has been questioned in athletic endeavors [28]. We did not compare hydration status between ultrasound and urine analysis, so we cannot comment on inter-measurement validity. Also, the summer of 2013 on Denali was unseasonably warm and mild with less than average storms. There was an average of 68% success rate of all climbers that year, as compared to an average of 50% success rate over the years prior [15]. Our data mirrored the average summit success for the mountain that year, so the results are likely generalizable to the general climbing population on Denali.

## Conclusions

Approximately half of the study participants were dehydrated, 57% by IVC-CI on ultrasound and 55% by urine specific gravity, but with no significant correlation of hydration status with subsequent summit success. Greater water-carrying capacity would allow a climber to increase hydration at times of high exertion and therefore may help with success in the high mountains. That being said, an *ad libitum *approach to hydration in the alpine environment is largely influenced by accessibility of drinking water. A simple and inexpensive educational intervention for mountaineers would be to carry more water and to impart an emphasis on hydration, which may contribute to improved mountaineering performance. Future studies on this subject could benefit from prospectively gathering data on fluid intake for the duration of a climb, with alternative measures of performance as well as other means of hydration assessment.
