# Correction: Hydration Status as a Predictor of High-altitude Mountaineering Performance

**DOI:** 10.7759/cureus.c7

**Published:** 2017-02-16

**Authors:** Eric Ladd, Katherine M Shea, Patrick Bagley, Sean Rundell, Paul S Auerbach, Elizabeth A Pirrotta, Ewen Wang, Grant S Lipman

**Affiliations:** 1 Department of Emergency Medicine, Stanford University School of Medicine; 2 University of New England College of Osteopathic Medicine, University of New England; 3 Department of Rehabilitation Medicine, University of Washington

**Title:** Hydration Status as a Predictor of High-altitude Mountaineering Performance

**Original, Incorrect Author List:** Eric Ladd, Katherine M. Shea, Patrick Bagley, Paul S. Auerbach, Elizabeth A. Pirrotta, Ewen Wang, Grant S. Lipman

**Original Citation:** Ladd E, Shea K M, Bagley P, et al. (December 07, 2016) Hydration Status as a Predictor of High-altitude Mountaineering Performance. Cureus 8(12): e918. doi:10.7759/cureus.918

The submitting author erroneously omitted the fourth author, Sean Rundell. This mistake was only just noticed by the authors. The author list should be corrected as follows. The original, incorrect author list will be changed.

**Corrected Author List: **Eric Ladd, Katherine M. Shea, Patrick Bagley, Sean Rundell, Paul S. Auerbach, Elizabeth A. Pirrotta, Ewen Wang, Grant S. Lipman

