# Correction: Hydration Status as a Predictor of High-altitude Mountaineering Performance

**DOI:** 10.7759/cureus.c10

**Published:** 2017-09-08

**Authors:** Eric Ladd, Katherine M Shea, Patrick Bagley, Paul S Auerbach, Elizabeth A Pirrotta, Ewen Wang, Grant Lipman

**Affiliations:** 1 Department of Emergency Medicine, Stanford University School of Medicine; 2 University of New England College of Osteopathic Medicine, University of New England; 3 School of Medicine, Stanford University

**Title:** Hydration Status as a Predictor of High-altitude Mountaineering Performance

**Original Author List:** Eric Ladd, Katherine M. Shea, Patrick Bagley, Paul S. Auerbach, Elizabeth A. Pirrotta, Ewen Wang, Grant S. Lipman

**Original Citation:** Ladd E, Shea K M, Bagley P, et al. (December 07, 2016) Hydration Status as a Predictor of High-altitude Mountaineering Performance. Cureus 8(12): e918. doi:10.7759/cureus.918

The submitting author originally reported that an author was missing. This was verified with the author in question (Sean Rundell) and he was added to the author list (at which point a correction was issued). We have now received word that this was a mistake and that the author was to be added to a different article. As such, we are removing Sean Rundell from the author list for this article.

